# CVIT expert consensus document on primary percutaneous coronary intervention (PCI) for acute myocardial infarction (AMI) update 2022

**DOI:** 10.1007/s12928-021-00829-9

**Published:** 2022-01-12

**Authors:** Yukio Ozaki, Hironori Hara, Yoshinobu Onuma, Yuki Katagiri, Tetsuya Amano, Yoshio Kobayashi, Takashi Muramatsu, Hideki Ishii, Ken Kozuma, Nobuhiro Tanaka, Hitoshi Matsuo, Shiro Uemura, Kazushige Kadota, Yutaka Hikichi, Kenichi Tsujita, Junya Ako, Yoshihisa Nakagawa, Yoshihiro Morino, Ichiro Hamanaka, Nobuo Shiode, Junya Shite, Junko Honye, Tetsuo Matsubara, Kazuya Kawai, Yasumi Igarashi, Atsunori Okamura, Takayuki Ogawa, Yoshisato Shibata, Takafumi Tsuji, Junji Yajima, Kaoru Iwabuchi, Nobuo Komatsu, Teruyasu Sugano, Masaru Yamaki, Shinichiro Yamada, Hiroaki Hirase, Yuusuke Miyashita, Fuminobu Yoshimachi, Masakazu Kobayashi, Jiro Aoki, Hirotaka Oda, Yoshiaki Katahira, Kinzo Ueda, Masami Nishino, Koichi Nakao, Ichiro Michishita, Takafumi Ueno, Taku Inohara, Shun Kohsaka, Tevfik F. Ismail, Patrick W. Serruys, Masato Nakamura, Hiroyoshi Yokoi, Yuji Ikari, The Task Force on Primary Percutaneous Coronary Intervention (PCI) of the Japanese Cardiovascular Interventional Therapeutics (CVIT)

**Affiliations:** 1 Department of Cardiology, Fujita Health University School of Medicine, Aichi, Japan; 2 Department of Cardiology, National University of Ireland, Galway (NUIG), Galway, Ireland; 3 Department of Cardiology, Academic Medical Center, University of Amsterdam, Amsterdam, The Netherlands; 4 Department of Cardiology, Sapporo Higashi Tokushukai Hospital, Sapporo, Japan; 5 Department of Cardiology, Aichi Medical University, Aichi, Japan; 6 Department of Cardiovascular Medicine, Chiba University Graduate School of Medicine, Chiba, Japan; 7 Department of Cardiovascular Medicine, Gunma University Graduate School of Medicine, Gunma, Japan; 8 Department of Cardiology, Teikyo University Hospital, Tokyo, Japan; 9 Division of Cardiology, Tokyo Medical University Hachioji Medical Center, Tokyo, Japan; 10 Gifu Heart Center, Gifu, Japan; 11 Cardiovascular Medicine, Kawasaki Medical School, Kurashiki, Japan; 12 Kurashiki Central Hospital, Kurashiki, Japan; 13 Heart Center, Saga Medical Center Koseikan, Saga, Japan; 14 Department of Cardiovascular Medicine, Graduate School of Medical Sciences, Kumamoto University, Kumamoto, Japan; 15 Department of Cardiology, Kitasato University Hospital, Sagamihara, Japan; 16 Division of Cardiovascular Medicine, Department of Internal Medicine, Shiga University of Medical Science, Otsu, Japan; 17 Department of Cardiology, Iwate Medical University Hospital, Morioka, Japan; 18 Cardiovascular Intervention Center, Rakuwakai Marutamachi Hospital, Kyoto, Japan; 19 Division of Cardiology, Hiroshima City Hiroshima Citizens Hospital, Hiroshima, Japan; 20 Cardiology Division, Osaka Saiseikai Nakatsu Hospital, Osaka, Japan; 21 Kikuna Memorial Hospital, Yokohama, Japan; 22 Toyohashi Heart Center, Toyohashi, Japan; 23 Chikamori Hospital, Kochi, Japan; 24 Sapporo-Kosei General Hospital, Sapporo, Japan; 25 Sakurabashi Watanabe Hospital, Osaka, Japan; 26 Division of Cardiology, The Jikei University School of Medicine, Tokyo, Japan; 27 Miyazaki Medical Association Hospital, Miyazaki, Japan; 28 Kusatsu Heart Center, Kusatsu, Japan; 29 The Cardiovascular Institute, Tokyo, Japan; 30 Osaki Citizen Hospital, Osaki, Japan; 31 Ota Nishinouchi Hospital, Fukushima, Japan; 32 Yokohama City University Hospital, Yokohama, Japan; 33 Nayoro City General Hospital, Nayoro, Japan; 34 Kitaharima Medical Center, Ono, Japan; 35 Takaoka Minami Heart Center, Takaoka, Japan; 36 Nagano Red Cross Hospital, Nagano, Japan; 37 Tokai University Hachioji Hospital, Tokyo, Japan; 38 Division of Cardiology, Mitsui Memorial Hospital, Tokyo, Japan; 39 Niigata City General Hospital, Niigata, Japan; 40 Department of Cardiology, Katta General Hospital, Shiroishi, Japan; 41 Takase Clinic, Takasaki, Japan; 42 Division of Cardiology, Osaka Rosai Hospital, Osaka, Japan; 43 Division of Cardiology, Saiseikai Kumamoto Hospital, Cardiovascular Center, Kumamoto, Japan; 44 Yokohama Sakae Kyosai Hospital, Yokohama, Japan; 45 Fukuoka Kinen Hospital, Fukuoka, Japan; 46 Keio University School of Medicine, Tokyo, Japan; 47 King’s College London & Guy’s and St Thomas’ Hospital NHS Foundation Trust, London, UK; 48 NHLI, Imperial College London, London, UK; 49 Division of Cardiovascular Medicine, Ohashi Medical Center, Toho University School of Medicine, Tokyo, Japan; 50 Cardiovascular Center, Fukuoka Sanno Hospital, Fukuoka, Japan; 51 Department of Cardiology, Tokai University School of Medicine, Isehara, Japan

**Keywords:** ST-segment elevation acute myocardial infarction (STEMI), Non-ST-segment elevation acute coronary syndrome (NSTE-ACS), Percutaneous coronary intervention (PCI), Thrombus aspiration, Optical coherence tomography (OCT), Dual antiplatelet therapy (DAPT)

## Abstract

Primary Percutaneous Coronary Intervention (PCI) has significantly contributed to reducing the mortality of patients with ST-segment elevation myocardial infarction (STEMI) even in cardiogenic shock and is now the standard of care in most of Japanese institutions. The Task Force on Primary PCI of the Japanese Association of Cardiovascular Interventional and Therapeutics (CVIT) society proposed an expert consensus document for the management of acute myocardial infarction (AMI) focusing on procedural aspects of primary PCI in 2018. Updated guidelines for the management of AMI were published by the European Society of Cardiology (ESC) in 2017 and 2020. Major changes in the guidelines for STEMI patients included: (1) radial access and drug-eluting stents (DES) over bare-metal stents (BMS) were recommended as a Class I indication, (2) complete revascularization before hospital discharge (either immediate or staged) is now considered as Class IIa recommendation. In 2020, updated guidelines for Non-ST-Elevation Myocardial Infarction (NSTEMI) patients, the followings were changed: (1) an early invasive strategy within 24 h is recommended in patients with NSTEMI as a Class I indication, (2) complete revascularization in NSTEMI patients without cardiogenic shock is considered as Class IIa recommendation, and (3) in patients with atrial fibrillation following a short period of triple antithrombotic therapy, dual antithrombotic therapy (e.g., DOAC and single oral antiplatelet agent preferably clopidogrel) is recommended, with discontinuation of the antiplatelet agent after 6 to 12 months. Furthermore, an aspirin-free strategy after PCI has been investigated in several trials those have started to show the safety and efficacy. The Task Force on Primary PCI of the CVIT group has now proposed the updated expert consensus document for the management of AMI focusing on procedural aspects of primary PCI in 2022 version.

## Introduction

In ST-segment elevation myocardial infarction (STEMI), primary PCI has been shown to contribute to the reduction of cardiac events, lead to earlier discharge, and is even effective in patients with cardiogenic shock [1–19]. It is now a standard of care in Japan. While there are a high number of available facilities providing primary PCI in Japan, there are no guidelines focusing on procedural aspect of standardized care, which may further improve the quality of our practice.

Updated guidelines for the management of acute myocardial infarction (AMI) were published by European Society of Cardiology (ESC) in 2017 and 2020 [20, 21]. Major changes in the guidelines for STEMI patients include recommendations for: (1) radial access and drug-eluting stent (DES) over bare-metal stent (BMS) as a Class I indication; and (2) complete revascularization before hospital discharge (either immediate or staged) as a Class IIa recommendation. Primary PCI is consistently recommended in the updated guidelines as well as the previous guidelines [22]. In the guidelines for NSTEMI patients, the followings were changed: (1) an early invasive strategy within 24 h is recommended in patients with NSTEMI as a Class I indication; (2) complete revascularization in NSTEMI patients without cardiogenic shock is considered as Class IIa recommendation; and (3) in patients with atrial fibrillation, following a short period of triple antithrombotic therapy (up to 1 week from the acute event), dual antithrombotic therapy (e.g., DOAC and single oral antiplatelet agent preferably clopidogrel) is recommended, with cessation of the antiplatelet after 6 to 12 months [21].

However, lags in device approval and differences in available medical therapy and mechanical support may prevent direct application of European guidelines to the Japanese population (Tables 1 and 2). Therefore, the Task Force on PCI of the Japanese CVIT society summarized the consensus for the management of AMI, mainly focusing on procedural aspects.

**Table 1:** Major differences in available medication and mechanical devices

	Europe	Japan
Glycoprotein IIb/IIIa inhibitors	Tirofiban, eptifibatide, and abciximab are available	GP IIb/IIIa inhibitors are not available
P2Y12 inhibitors	The preferred P2Y12 inhibitors are prasugrel [60 mg loading dose and 10 mg maintenance dose once daily per os (p.o.)] or ticagrelor (180 mg p.o. loading dose and 90 mg maintenance dose twice daily)	Both prasugrel and ticagrelor are available, but the dose in prasugrel is different. [20 mg loading dose and 3.75 mg maintenance dose once daily per os]
Mechanical LV assist devices	Intra-cardiac axial flow pump (i.e., Impella) and intra-aortic balloon pump are available	Intra-aortic balloon pumps are still in use. Intra-cardiac axial flow pumps are used in selected institutions but are not widely available

**Table 2:** Major CE-approved DES and their availability in Japan

DES	Stent platform	Polymer coating	Drug	Availability in Japan
Based on durable polymer coatings				
DESyne Nx	Cobalt–chrome	PBMA	Novolimus	No
Promus element	Platinum–chrome	PBMA and PVDF-HFP	Everolimus	Yes
Resolute	Cobalt–chrome	PBMA, PHMA, PVP, and PVA	Zotarolimus	Yes
STENTYS	Nitinol	PSU and PVP	Paclitaxel	No
Xience	Cobalt–chrome	PBMA and PVDF-HFP	Everolimus	Yes
Based on biodegradable polymer coatings				
Axxess	Nitinol	PDLLA	Biolimus A9	No
Biomatrix	Stainless steel	PDLLA	Biolimus A9	No
BioMime	Cobalt–chrome	PLLA and PLGA	Sirolimus	No
Combo	Stainless steel	PDLLA and PLGA + Additional coating with anti-CD34	Sirolimus	No
DESyne BD	Cobalt–chrome	PLLA	Novolimus	No
Infinnium	Stainless steel	PLLA, PLGA, PCL, and PVP	Paclitaxel	No
MiStent	Cobalt–chrome	PLGA	Crystalline sirolimus	No
Nobori	Stainless steel	PDLLA	Biolimus A9	Yes
Orsiro	Cobalt–chrome	PLLA	Sirolimus	Yes
Supralimus core	Cobalt–chrome	PLLA, PLGA, PCL, and PVP	Sirolimus	No
Synergy	Platinum–chrome	PLGA	Everolimus	Yes
Ultimaster	Cobalt–chrome	PDLLA and PCL	Sirolimus	Yes
Yukon choice PC	Stainless steel	PDLLA	Sirolimus	No
Supraflex Cruz	Cobalt–chrome	PLLA, PLGA, and PVP	Sirolimus	No
BuMA supreme	Cobalt–chrome	PLGA	Sirolimus	No
Polymer-free				
Amazonia Pax	Cobalt–chrome	–	Paclitaxel	No
BioFreedom	Stainless steel	–	Biolimus A9	Yes
Cre8	Cobalt–chrome	–	Sirolimus	No
Yukon Choice PF	Stainless steel	–	Sirolimus	No

*PBMA* polyn-butyl methacrylate, *PCL* poly-caprolactone, *PDLLA* poly-D,L-lactic acid, *PHMA* poly-hexyl methacrylate, *PLCL* poly-L-lactide-co-caprolactone, *PLGA* poly-D,L-lactide-co-glycolide, *PLLA* poly-L-lactic acid, *PVP* polyvinylpyrrolidone, *PVA* polyvinyl acetate, *PVDF-HFP* poly-vinylidene fluoride-co-hexafluoropropylene, *PSU* polysulphone

### Specific differences between Japan and Europe

Glycoprotein (GP) IIb/IIIa inhibitors are not available in Japan whereas thrombus aspiration is still a choice of strategy of treatment of AMI.

Currently preferred oral P2Y12 inhibitors in acute coronary syndrome in Europe are prasugrel and ticagrelor. Until recently, ticagrelor was not available in Japan, which was approved in 2016 and put on the market in February, 2017, with a 7-year lag from the approval in Europe. In addition, dose difference in P2Y12 inhibitors between Japan and Europe may cause different antithrombotic benefit/bleeding risk profile. Intravenous cangrelor and subcutaneous selatogrel are not approved in Japan, while its use may be considered in patients not pre-treated with oral P2Y12 inhibitors at the time of PCI or in those who are considered unable to absorb oral agents.

LV assist devices and ECMO are increasingly popular managing patients with cardiogenic shock in Europe although they have not been sufficiently evaluated in clinical trials, while the use of IABP has not met prior expectations of benefit [23, 24]. In contrast, in Japan, left ventricular assist devices (LVADs: i.e., intra-cardiac axial flow pumps and arterial-venous extracorporeal membrane oxygenation) are not widely available, and we continue to largely rely on intra-aortic balloon pumps as a mechanical support.

Regarding intravascular imaging devices, intravascular ultrasound (IVUS) and optical coherence tomography (OCT) during PCI are routinely reimbursed in Japan. In contrast to the situation in Europe, their use is not restricted in selected cases such as unprotected left main lesions or stent failure.

In terms of data derived from Japanese population, there are several registries and databases including patients with AMI in Japan such as J-MINUET [25–28], PACIFIC [29], Tokyo CCU network registry [30], JAMIR [31–35], and JROAD [36–38]. CVIT has been working on the J-PCI registry [39–42], the largest database of patients who underwent PCI in Japan. The current demographics, lesion and procedural characteristics in patients with AMI in Japan can be identified in the J-PCI registry including 253,228 patients treated in 1113 institutions from January 2019 to December 2019 (Tables 3 and 4).

**Table 3:** Demographics of patients with STEMI and NSTEMI from J-PCI registry

	Overall MI	STEMI	NSTEMI	*p* value
	(*N* = 59,020)	(*n* = 44,329)	(*n* = 14,691)	
Age (years), mean (SD)	69.86 (12.84)	69.49 (12.94)	70.95 (12.49)	< 0.001
Female	14,200 (24.1)	10,686 (24.1)	3514 (23.9)	0.654
Cardiogenic shock	6798 (11.5)	5570 (12.6)	1228 (8.4)	< 0.001
Risk factors				
Smoker (current and within 1 yr)	21,763 (36.9)	16,720 (37.7)	5043 (34.3)	< 0.001
Diabetes mellitus	21,525 (36.5)	15,547 (35.1)	5978 (40.7)	< 0.001
Hypertension	40,711 (69.0)	29,828 (67.3)	10,883 (74.1)	< 0.001
Hypercholesterolemia	34,823 (59.0)	25,546 (57.6)	9277 (63.1)	< 0.001
History of:				
Previous MI	7008 (11.9)	4344 (9.8)	2664 (18.1)	< 0.001
Peripheral vascular disease	2184 (3.7)	1331 (3.0)	853 (5.8)	< 0.001
Previous PCI	9365 (15.9)	5871 (13.2)	3494 (23.8)	< 0.001
Previous CABG	824 (1.4)	384 (0.9)	440 (3.0)	< 0.001
Heart failure	4503 (7.6)	2650 (6.0)	1853 (12.6)	< 0.001
Renal insufficiency	10,506 (17.8)	7219 (16.3)	3287 (22.4)	< 0.001
Hemodialysis	1745 (3.0)	958 (2.2)	787 (5.4)	< 0.001
Chronic lung disease (COPD)	1453 (2.5)	1023 (2.3)	430 (2.9)	< 0.001
Door to balloon time				
Min, median (IQR: 25th, 75th)	NA	70 (54, 90)	NA	NA
Antiplatelet prescribed before or at procedure				
Type of antiplatelet agent				
Aspirin	47,403 (80.3)	35,165 (79.3)	12,238 (83.3)	< 0.001
Clopidogrel	7283 (12.3)	4488 (10.1)	2795 (19.0)	< 0.001
Prasugrel	36,465 (61.8)	27,990 (63.1)	8475 (57.7)	< 0.001
Ticagrelor	65 (0.1)	34 (0.1)	31 (0.2)	< 0.001
Dual antiplatelet therapy				
Aspirin + clopidogrel	6736 (11.4)	4145 (9.4)	2591 (17.6)	< 0.001
Aspirin + ticagrelor	60 (0.1)	30 (0.1)	30 (0.2)	< 0.001
Aspirin + prasugrel	35,912 (60.8)	27,566 (62.2)	8346 (56.8)	< 0.001
Single antiplatelet therapy	5574 (9.4)	4039 (9.1)	1535 (10.4)	< 0.001
Oral anticoagulant prescribed before or at procedure				
Warfarin	887 (1.5)	615 (1.4)	272 (1.9)	< 0.001
DOAC	1442 (2.4)	924 (2.1)	518 (3.5)	< 0.001
In-hospital mortality	3097 (5.2)	2526 (5.7)	571 (3.9)	< 0.001

Data are counts (percentage) unless otherwise specified.

*CABG* coronary artery bypass grafting, *IQR* interquartile range, *MI*  myocardial infarction, *NSTEMI* non-ST-elevation myocardial infarction, *PCI* percutaneous coronary intervention, *STEMI* ST-elevation myocardial infarction

**Table 4:** Lesion and procedural characteristics in STEMI and NSTEMI from J-PCI registry

	Overall MI	STEMI	NSTEMI	*p* value
	(*N* = 59,020)	(*n* = 44,329)	(*n* = 14,691)	
Lesion characteristics				
Lesion location:				
LAD/left main	31,371 (53.2)	23,961 (54.1)	7410 (50.4)	< 0.001
LCX	11,704 (19.8)	6625 (14.9)	5079 (34.6)	< 0.001
RCA	22,586 (38.3)	18,194 (41.0)	4392 (29.9)	< 0.001
Bypass graft	159 (0.3)	74 (0.2)	85 (0.6)	< 0.001
Restenotic lesion	2824 (4.8)	1830 (4.1)	994 (6.8)	< 0.001
Procedure details				
Approach				< 0.001
Transfemoral	15,425 (26.1)	12,305 (27.8)	3120 (21.2)	
Transradial	41,859 (70.9)	30,831 (69.6)	11,028 (75.1)	
Others (e.g., brachial)	1736 (2.9)	1193 (2.7)	543 (3.7)	
Thrombus aspiration	24,915 (42.2)	22,042 (49.7)	2873 (19.6)	< 0.001
Distal protection	3114 (5.3)	2638 (6.0)	476 (3.2)	< 0.001
Stent characteristics				
DES	50,572 (85.7)	38,435 (86.7)	12,137 (82.6)	< 0.001
Mechanical assist device				
IABP	6367 (10.8)	5128 (11.6)	1239 (8.4)	< 0.001
PCPS	1589 (2.7)	1285 (2.9)	304 (2.1)	< 0.001
Impella	263 (0.4)	222 (0.5)	41 (0.3)	0.001
TIMI flow post-procedure				
Flow 3	57,964 (98.2)	43,596 (98.3)	14,368 (97.8)	< 0.001

Data are counts (percentage)

*BMS* bare-metal stent, *DES* drug-eluting stent, *LAD* left anterior descending artery, *LCx* left circumflex artery, MI = myocardial infarction, *NSTEMI* non ST-elevation myocardial infarction, *RCA*  right coronary artery, *STEMI* ST-elevation myocardial infarction, *TIMI* thrombolysis in Myocardial Infarction

## Primary PCI in STEMI, early invasive vs. conservative strategy in NSTEMI

In ST-segment elevation myocardial infarction, primary PCI has been shown to contribute high revascularization success rates, less cardiac events, earlier discharge, and is even effective in patients with cardiogenic shock [1–19] and consistently recommended by European [20], American [43], and Japanese guidelines.

Meta-analysis based on individual patient data from three studies that compared a routine invasive against a selective invasive strategy revealed lower rates of death and myocardial infarction at 5-year follow-up (HR = 0.81, 95% CI 0.71–0.93; *p* = 0.002), with the most pronounced difference in high-risk patients [44]. Age, diabetes, previous myocardial infarction, ST-segment depression, hypertension, body mass index (< 25 kg/m^2^ or > 35 kg/m^2^) and treatment strategy were found to be independent predictors of death and myocardial infarction during follow-up. The results supported a routine invasive strategy but highlight the importance of risk stratification in the decision-making process as is recommended in the present guidelines [21, 23].

However, in the ICTUS trial, in which 1,200 patients with Non-ST-Elevation Acute Coronary Syndrome (NSTE-ACS) (NSTE-ACS) and an elevated cardiac troponin T were randomized to an early invasive strategy versus a selective invasive strategy, 10-year death or spontaneous MI was not statistically different between the 2 groups (33.8% vs. 29.0%, HR 1.12, 95% CI 0.97–1.46; *p* = 0.11). In addition, the 15-year follow-up of the FRISC-II study showed a significant 18-month postponement of the occurrence of death or next MI and 37 months postponement of re-hospitalization for ischemic heart disease, but similar mortality with either strategy [45].

Although the long-term benefit of an early invasive strategy is unclear, the timing of angiography and revascularization should be based on patient risk profile, considering the significant difference between early and delayed strategies in short-term outcome.

In the VERDICT trial, 2147 patients with NSTE-ACS were randomized to invasive coronary angiography within 12 h or standard invasive care within 48–72 h [46]. Overall, early invasive coronary angiography did not improve the primary endpoint at 5 years (all-cause death, nonfatal AMI, hospital admission for refractory myocardial ischemia, or hospital admission for heart failure; HR 0.92, 95% CI 0.78–1.08). However, in patients with a GRACE risk score > 140, early invasive coronary angiography significantly reduced the primary endpoint (HR 0.81, 95% CI 0.66–0.99). In addition, early invasive coronary angiography had some benefits in patients with troponin elevation (i.e., NSTEMI) and ST-T change (HR 0.85, 95% CI 0.71–1.01; and HR 0.80, 95% CI 0.63–1.01, respectively).

GRACE risk score was applied to the patients with acute coronary syndrome (ACS) in the Tokyo CCU (cardiovascular care unit) Network Database. A total of 9,460 patients with ACS hospitalized at 67 Tokyo CCUs were retrospectively reviewed and there was a strong correlation between the GRACE risk score and in-hospital mortality for patients with STEMI or NSTEMI (*r* = 0.99, *p* < 0.001); however, the correlation was not significant for patients with unstable angina (*r* = 0.35, *p* = 0.126). We recommend the use of the GRACE score to identify high-risk patients with AMI [47].

Recently a chronic kidney disease (CKD) study from J-MINUET examining the impact of CKD on long-term outcomes in 3,281 Japanese patients with AMI demonstrated that 3-year mortality and MACE significantly deteriorated from 5.09% and 15.8% in no CKD through 16.3% and 38.2% in moderate CKD to 36.7% and 57.9% in severe CKD, respectively (*p* < 0.0001) [48]. CKD remains a useful predictor of in-hospital and 3-year mortality as well as MACE after AMI in the modern PCI and medical therapy era [48].

In cases of cardiac arrest without STEMI, the COACT (Coronary angiography after cardiac arrest) trial compared immediate angiography with an intent to revascularize with delayed angiography in patients who successfully resuscitated after out-of-hospital cardiac arrest and had no signs of STEMI [49]. Immediate angiography did not reduce death, compared to delayed angiography [50].

### Recommendations

Primary PCI of the infarct-related artery (IRA) is indicated in STEMI.

#### In case of NSTEMI

Urgent coronary angiography (< 2 h) is recommended in patients at very high risk (refractory angina, ST changes in more than 6 leads, with associated heart failure, mechanical complication, cardiogenic shock, life-threatening arrhythmias, or hemodynamic instability).

An early invasive strategy (< 24 h) is recommended in patients with an established NSTEMI diagnosis.

## Practical recommendation for primary PCI

- Loading dose DAPT

Prasugrel and ticagrelor reduce ischemic events and mortality in ACS patients compared to clopidogrel and are recommended by current guidelines [20, 21, 51].

In TRITON-TIMI 38, 13,608 patients with ACS with scheduled PCI were randomized to either prasugrel or clopidogrel. Prasugrel therapy was associated with significantly reduced rates of ischemic events, including stent thrombosis, but with an increased risk of major bleeding, including fatal bleeding. Overall mortality did not differ significantly between the treatment groups [51]. In the Japanese population, the PRASFIT-ACS study was conducted to confirm the efficacy and safety of prasugrel at loading/maintenance doses of 20/3.75 mg [52]. Japanese patients (*n* = 1363) with ACS undergoing PCI were randomized to either prasugrel (20 mg for loading/3.75 mg for maintenance) or clopidogrel (300 mg for loading/75 mg for maintenance). The incidence of MACE at 24 weeks was 9.4% in the prasugrel group and 11.8% in the clopidogrel group (risk reduction 23%, hazard ratio 0.77, 95% confidence interval 0.56–1.07). The incidence of non-coronary artery bypass graft-related major bleeding was similar in both groups (1.9% vs. 2.2%). The results were similar to TRITON-TIMI 38 with a low risk of clinically serious bleeding in Japanese ACS patients.

Regarding ticagrelor, clinical outcomes in a large real-world post-ACS population were studied in a Swedish prospective cohort study of 45,073 ACS patients who were discharged on ticagrelor (*N* = 11,954) or clopidogrel (*N* = 33,119) [53]. The risk of the primary outcome (composite of all-cause death, readmission with Ml or stroke) with ticagrelor vs. clopidogrel was 11.7% vs. 22.3% [adjusted HR (HR) 0.85 (95% Cl: 0.78–0.93)], risk of death 5.8% vs. 12.9% (adjusted HR 0.83 [0.75–0.921], and risk of MI 6.1% vs. 10.8% (adjusted HR 0.89 [0.78–1.011] at 24 months. Re-admission rates for bleeding with ticagrelor versus clopidogrel were similar. Both ticagrelor and clopidogrel post-ACS was associated with a lower risk of death, Ml, or stroke, as well as death alone. Risk of bleeding was higher with ticagrelor [53]. These real-world outcomes are consistent with the results of the landmark PLATO (Platelet Inhibition and Patient Outcomes) trial [54].

The ISAR-REACT 5 trial compared prasugrel plus aspirin vs. ticagrelor plus aspirin in 4,018 ACS patients [55]. The trial demonstrated that treatment with prasugrel, compared to ticagrelor, significantly reduced the composite rate of death, MI, or stroke (6.9% vs. 9.3%, *p* = 0.006) without any increase in bleeding complications (4.8% vs. 5.4%, *p* = 0.46).

Both prasugrel and ticagrelor are available for clinical use in Japan. The recommended dose of prasugrel is the same as in Europe and United Sates of America, while the dose of prasugrel was reduced according to the PLASFIT-ACS study in Japan [52] (EU: 60 mg loading dose and 10 mg maintenance dose once daily; Japan: 20 mg loading dose and 3.75 mg maintenance dose once daily) (Table 1).

### Recommendations

A potent P2Y12 inhibitor (prasugrel or ticagrelor) as well as clopidogrel especially in patients having OAC or DOAC are recommended before or at latest at the time of PCI.

Recommended dose of prasugrel: 20 mg loading dose and 3.75 mg maintenance dose once daily per os (p.o.).

Recommended dose of ticagrelor: 180 mg p.o. loading dose and 90 mg maintenance dose twice daily.

b. Anticoagulation during PCI

According to the 2017 ESC STEMI and 2020 ESC NSTE-ACS Guidelines, routine use of unfractionated heparin (UFH) is recommended as a class I recommendation and routine use of enoxaparin or bivalirudin during primary PCI is a class IIa or IIb recommendation [20, 21].

There has been no placebo-controlled trial evaluating UFH in primary PCI, but there is a large body of experience with this agent. Dosage should follow standard recommendations for PCI (i.e., initial bolus 70–100 U/kg). There are no robust data recommending the use of activated clotting time to tailor dose or monitor UFH, and if activated clotting time is used, it should not delay recanalization of the artery.

An intravenous bolus of enoxaparin 0.5 mg/kg was compared with UFH in the ATOLL randomized trial including 910 STEMI patients [56]. The primary composite endpoint of 30-day death, MI, procedural failure, or major bleeding was not significantly reduced by enoxaparin (17% relative risk reduction, *p* = 0.063), but there was a reduction in the composite main secondary endpoint of death, recurrent MI or ACS, or urgent revascularization. Importantly, there was no evidence of increased bleeding following the use of enoxaparin over UFH. In a meta-analysis of 23 PCI trials (30,966 patients, 33% primary PCI), enoxaparin was associated with a significant reduction in death compared to UFH. This effect was particularly significant in the primary PCI context and was associated with a reduction in major bleeding [57]. In Japan, enoxaparin is approved only for subcutaneous administration and is practically difficult to use during PCI.

A meta-analysis comparing bivalirudin with UFH with or without planned use of GP IIb/IIIa inhibitors in patients with STEMI trials showed no mortality advantage with bivalirudin and a reduction in the risk of major bleeding, but at the cost of an increased risk of acute stent thrombosis [58]. In the recent MATRIX trial including 7213 ACS patients (56% with STEMI), bivalirudin did not reduce the incidence of the primary endpoint (composite of death, MI, or stroke) compared to UFH. Bivalirudin was associated with lower total and cardiovascular mortality, lower bleeding, and more definite stent thrombosis [59]. A post hoc analysis suggested that prolonging bivalirudin with a full-PCI dose after PCI was associated with the lowest risk of ischemic and bleeding events, which is in accordance with the current labeling of the drug [59]. Bivalirudin could be considered in STEMI, especially in patients at high bleeding risk [60–62]. Bivalirudin is recommended for patients with heparin-induced thrombocytopenia.

After the publication of the 2017 ESC guidelines, the VALIDATE-SWEDEHEART (Bivalirudin versus Heparin in ST-Segment and Non-ST-Segment Elevation Myocardial Infarction in Patients on Modern Antiplatelet Therapy in the Swedish Web System for Enhancement and Development of Evidence-based Care in Heart Disease Evaluated according to Recommended Therapies Registry Trial) multicenter, randomized, registry-based trial was published [63]. Patients with either ST-segment elevation MI (*N* = 3005) or non-ST-segment elevation MI (*N* = 3001) undergoing PCI and receiving a potent P2Y12 inhibitor (ticagrelor, prasugrel, or cangrelor) without the planned use of glycoprotein IIb/IIIa inhibitors were randomly assigned to receive bivalirudin or heparin during PCI, performed predominantly with the use of radial artery access. The primary composite endpoint (death from any cause, MI, or major bleeding during 180 days of follow-up) occurred in 12.3% of the patients in the bivalirudin group and in 12.8% in the heparin group (HR 0.96, 95% CI 0.83 to 1.10; *p* = 0.54). The results were consistent between patients with ST-segment elevation MI and those with non-ST-segment elevation MI and across other major subgroups. There was no difference between groups in MI, major bleeding, definite stent thrombosis or mortality. This study shows overall clinical non-inferiority for use of bivalirudin or heparin during PCI for ACS, along with increased cost with use of bivalirudin. Thus, the use of bivalirudin during PCI was downgraded to a class IIb recommendation. Consistent with these findings, the current uptake of bivalirudin in Europe is very low. Bivalirudin remains unavailable in Japan with no evaluation by clinical trials.

Glycoprotein (GP) IIb/IIIa inhibitors are the strongest antiplatelet agents currently available in Europe and in the US, but remain unavailable in Japan. There are three different compounds, namely abciximab, tirofiban, and eptifibatide. However, procedural use of abciximab plus unfractionated heparin (UFH) showed no benefit compared to bivalirudin [60]. In Japan, JEPPORT a randomized placebo-controlled trial (*n* = 973), abciximab did not show efficacy in reducing the primary endpoint (30-day post-PCI coronary events: death, MI or urgent revascularization) [64]. However, using GP IIb/IIIa inhibitors as bail-out therapy in the event of angiographic evidence of a large thrombus, slow or no reflow, and other thrombotic complications is reasonable, as recommended in 2017 ESC guidelines [20], although this strategy has not been tested in a randomized trial. Overall, there is no evidence to recommend the routine use of GP IIb/IIIa inhibitors for primary PCI.

### Recommendations

Anticoagulation is recommended for all the patients in addition to antiplatelet therapy during primary PCI.

Routine use of UFH is recommended.

c. Approach (femoral vs. radial)

Over recent years, several studies have provided robust evidence in favor of the radial approach as the default access site in ACS patients undergoing primary PCI by experienced radial operators [65, 66]. In the Minimizing Adverse Hemorrhagic Events by TRansradial Access Site and Systemic Implementation of angioX (MATRIX) program, patients were randomized to radial or femoral access, stratified by STEMI (2,001 radial, 2,009 femoral) and NSTE-ACS (2,196 radial, 2,198 femoral). MACE occurred in 121 (6.1%) STEMI patients with radial access vs. 126 (6.3%) patients with femoral access [rate ratio (RR) = 0.96, 95% CI = 0.75–1.24; *p* = 0.76] and in 248 (11.3%) NSTE-ACS patients with radial access vs. 303 (13.9%) with femoral access (RR = 0.80, 95% CI = 0.67–0.96; *p* = 0.016) (Pint = 0.25). MACE occurred in 142 (7.2%) STEMI patients with radial access and in 165 (8.3%) patients with femoral access (RR = 0.86, 95% CI = 0.68–1.08; *p* = 0.18) and in 268 (12.2%) NSTE-ACS patients with radial access compared with 321 (14.7%) with femoral access (RR = 0.82, 95% CI = 0.69–0.97; *p* = 0.023) (P_int_ = 0.76). All-cause mortality and access site-actionable bleeding favored radial access irrespective of ACS type (*P*_interaction_ = 0.11 and *P*_interaction_ = 0.36, respectively) [67]. Radial as compared with femoral access was shown to have consistent benefit across the whole spectrum of patients with ACS, resulting in upgrading of the recommendation to a class I indication in the 2017 and 2020 ESC guidelines.

In Japan, the TEMPURA trial randomized patients with AMI undergoing primary PCI to transradial coronary intervention (TRI) group (*n* = 77) and transfemoral coronary intervention (TFI) group (*n* = 72) [68]. The success rate of reperfusion and the incidence of in-hospital MACE were similar in both groups (96.1% and 5.2% vs. 97.1% and 8.3% in TRI and TFI groups, respectively). In a substudy of PRASFIT-ACS including ACS patients with prasugrel, rates of periprocedural bleeding, bleeding not related to CABG, and puncture site bleeding were consistently lower in the TRI group than in the TFI group [69]. More recently, in a report from the CREDO-Kyoto AMI registry was published [70]. 3662 STEMI patients who had primary PCI by TRI (N = 471) or TFI (*N* = 3191) were analyzed. The prevalence of hemodynamically compromised patients (Killip II–IV) was significantly less in TRI group than in TFI group (19 vs. 25%, *p* = 0.002). Cumulative 5-year incidences of death/MI/stroke and major bleeding were not significantly different between the TRI and TFI groups (26.7 vs. 25.9%, log-rank *p* = 0.91, and 11.3 vs. 11.5%, log-rank *p* = 0.71, respectively). After adjustment for confounders, the risks of the TRI or TFI group were not significant for both death/MI/stroke [Hazard ratio (HR) 1.15, 95% confidence interval (CI) 0.83–1.59, *p* = 0.41] and major bleeding (HR 1.29, 95% CI 0.77–2.15, *p* = 0.34), leading to the conclusion that clinical outcomes of transradial approach were not different from those of transfemoral approach in primary PCI for STEMI in the real-world practice.

### Recommendations

Radial access is recommended over femoral access if performed by an experienced radial operator.

d. Thrombus aspiration

While it has been well recognized that thrombus formation caused by plaque rupture, plaque erosion and calcified nodule play a crucial role in the mechanism of ACS, the reduction of thrombus burden can theoretically be effective therapy for AMI [71–75]. However, in the guidelines released by the European Society of Cardiology in 2017 on the management of patients with STEMI, routine thrombus aspiration was downgraded from IIa to III (not recommended).

A pooled analysis of individual patient data from three large randomized trials (TAPAS [Thrombus Aspiration During Percutaneous Coronary Intervention in Acute Myocardial Infarction], TASTE [Thrombus Aspiration in ST-Elevation Myocardial Infarction in Scandinavia], and TOTAL [Trial of Routine Aspiration Thrombectomy With PCI Versus PCI Alone in Patients With STEMI]) provided novel insights about thrombus aspiration for ST-elevation MI [76]. By including 18,306 patients, the study did not show a significant reduction in cardiovascular death when thrombus aspiration was compared with standard therapy. There were also no differences between thrombus aspiration and no thrombus aspiration with respect to stroke or transient ischemic attack, recurrent MI, stent thrombosis, heart failure or target vessel revascularization [77]. Although routine use of mechanical thrombus aspiration is no longer recommended, prior safety concerns regarding the risk of stroke could not be confirmed. However, because a trend toward reduced cardiovascular death and increased stroke or transient ischemic attack was found in the subgroup of patients with high thrombus burden, future studies may want to investigate improved thrombus aspiration technologies in this high-risk subgroup.

In contrast to the studies mentioned above, earlier studies had shown a potential benefit for thrombus aspiration in primary PCI [78, 79].

### Evidence from Japan

There are several studies in Japan showing the benefit of thrombus aspiration in primary PCI.

In the VAMPIRE study, patients with STEMI were randomized to primary PCI with (*n* = 180) or without (*n* = 175) upfront thrombus aspiration [80]. There was a trend towards a lower incidence of slow or no reflow (primary endpoint defined as a Thrombolysis In Myocardial Infarction flow grade < 3) in patients treated with aspiration versus conventional primary PCI (12.4% vs. 19.4%, *p* = 0.07). The rate of myocardial blush grade 3 was higher in the aspiration group (46.0% vs. 20.5%, *p* < 0.001). Aspiration was most effective in patients presenting after 6 h of symptoms onset (slow flow rate: 8.1% vs. 37.6%, *p* = 0.01). Patients presenting late after STEMI appear to benefit the most from thrombectomy.

In an observational study (*n* = 3913) by Nakatani et al. [81], thrombus aspiration was associated with a lower 30-day mortality rate in selected patients with high TIMI risk scores, an age > or = 70 years, diabetes mellitus, or stenting adjusted for baseline characteristics.

In the latest guidelines of Japanese Circulation Society, thrombus aspiration in primary PCI was recommended as a class IIa indication with level of evidence B. Accordingly, thrombus aspiration is performed frequently in primary PCI in Japan. A comparison of specifications of aspiration device is tabulated in Table 5. From a practical view point, aspiration performance, trackability, and pushability are of importance when choosing an aspiration catheter [82].

**Table 5:** Thrombus aspiration catheters commercially available in Japan

Company	Product name	Guiding catheter compatibility	Guidewire compatibility (inch)	Catheter length (cm)	Wire lumen length (mm)	Distal outer diameter (mm)	Distal inner diameter (mm)	Proximal outer diameter (mm)	Proximal inner diameter (mm)	Length of hydrophilic coating (cm)	Shape of aspiration lumen	Stylet
Terumo	Eliminate + SL	6 Fr	0.014	140	90	1.70	0.98	1.40	1.05	40	Circle	No
	Eliminate + XL	6 Fr	0.014	140	90	1.75	1.10	1.40	1.15	40	Circle	Yes
		7 Fr	0.014	140	90	1.98	1.30	1.60	1.35	40	Circle	Yes
Medtronic	Export Advance	6 Fr	0.014	140	200	1.70	1.09	1.37	1.12	38	Circle	Yes
Kaneka	Thrombuster II	6 Fr	0.014	140	10	1.30	1.00	1.30	1.10	30	Circle	Yes
		7 Fr	0.014	140	10	1.50	1.20	1.53	1.32	30	Circle	Yes
		8 Fr	0.014	140	10	1.73	1.35	1.73	1.50	30	Circle	Yes
		9 Fr	0.014	140	10	2.00	1.50	2.00	1.75	30	Circle	Yes
	Thrombuster III SLa	6 Fr	0.014	140	120	1.35	1.00	1.35	1.00	30	Circle	No
		7 Fr	0.014	140	120	1.55	1.25	1.55	1.25	30	Circle	No
	Thrombuster III GRa	6 Fr	0.014	140	120	1.35	1.16	1.35	1.16	30	Circle	Yes
		7 Fr	0.014	140	120	1.55	1.36	1.55	1.36	30	Circle	Yes
Nipro	TVAC II	6 Fr	0.014	140	240	1.77	0.95	1.40	0.95	24	Circle	Yes^a^
		7 Fr	0.014	140	240	1.90	1.18	1.60	1.18	24	Circle	Yes^a^
	TVAC SOFT	6 Fr	0.014	135	250	1.50	NA	1.30	NA	25	Crescent	No
		7 Fr	0.014	135	250	1.50	NA	1.50	NA	25	Crescent	No
		8 Fr	0.014	135	250	1.80	NA	1.80	NA	25	Crescent	No
Goodman	Rebirth Pro 2	6Fr	0.014	136	220	1.35 × 1.62	1.09	1.38	1.11	60	Circle	Yes
		7 Fr	0.014	136	220	1.60 × 1.90	1.34	1.58	1.25	60	Circle	Yes

^a^There is TVAC II with or without stylet

Anzai et al. reported that thrombus aspiration facilitates direct stenting without increasing the cost of treatment [83]. Thrombus aspiration can be considered followed by direct stenting, which will be discussed later.

### Recommendations

Thrombus aspiration can be considered in primary PCI in the absence of GP IIb/IIIa inhibitors.

e. Distal protection

The benefit of distal protection using filter device or occlusion balloon has not been confirmed [84, 85]. However, the use of distal protection devices can be considered when plaque burden is large and there is a high possibility of distal embolism or no reflow.

### Evidences from Japan

Isshiki et al. reported initial clinical experience with Filtrap™ distal protection filter [86]. Filtrap™ was successfully delivered and deployed distal to the lesion in 13 of 14 patients (93%). Embolic debris was entrapped in 8 (62%) of these cases. All the patients were free from in-hospital events except for one patient with a large anterior AMI who received emergency surgery due to a free wall cardiac rupture. In the ASPARAGUS trial (*n* = 341), patients with AMI were randomized to either stenting with or without GuardWire Plus™ [87]. The rates of slow flow and no reflow immediately after PCI were 5.3 and 11.4% in the GuardWire Plus and control groups, respectively (*p* = 0.05). Blush score 3 acquisition rates immediately after PCI were 25.2 and 20.3% in the GuardWire Plus and control groups, respectively (*p* = 0.26), and the rates at 30 days after PCI were 42.9 and 30.4%, respectively (*p* = 0.035). In the CANARY pilot trial, near-infrared spectroscopy and intravascular ultrasound were performed at baseline, and lesions with a maximal lipid core burden index over any 4-mm length (maxLCBI_4mm_) ≥ 600 were randomized to PCI with versus without a distal protection filter [88]. Among 31 randomized lesions with maxLCBI_4mm_ ≥ 600, there was no difference in the rates of periprocedural MI with versus without the use of a distal protection filter (35.7% vs. 23.5%, *p* = 0.69). More recently, the VAMPIRE 3 trial randomized 200 ACS patients who had attenuated plaque with a longitudinal length of ≥ 5 mm by pre-PCI intravascular ultrasound to either distal protection (DP) by filter or conventional treatment (CT) [89]. The primary endpoint of no-reflow phenomenon occurred in 26.5% of the DP group (*n* = 98) and 41.7% of the CT group (*n* = 96; *p* = 0.0261) and the corrected TIMI frame count after revascularization was significantly lower in the DP group (23 vs 30.5; *p* = 0.0003). In addition, the incidence of in-hospital adverse cardiac events was significantly lower in the DP group than in the CT group (0% vs 5.2%; *p* = 0.028). Future studies may further elucidate whether distal protection is beneficial in selected patient.

In contrast, distal embolic protection during PCI of saphenous vein grafts was confirmed in a multicenter randomized controlled trial. In the SAFER randomized trial, a composite of death, myocardial infarction, emergency bypass, or target lesion revascularization by 30 days was observed in 16.5% in the control group and 9.6% in the embolic protection device (*p* = 0.004) [90]. This 42% relative reduction in major adverse cardiac events was driven by myocardial infarction (8.6% versus 14.7%, *p* = 0.008) and “no-reflow” phenomenon (3% versus 9%, *p* = 0.02). Clinical benefit was seen even when platelet glycoprotein IIb/IIIa receptor blockers were administered (61% of patients), with composite end points occurring in 10.7% of protection device patients versus 19.4% of control patients (*p* = 0.008). This study demonstrated the importance of prevention of distal embolization in saphenous vein graft.

Currently available filter devices in Japan are tabulated in Table 6.

**Table 6:** Filter devices for distal protection commercially available in Japan

Company	Product name	Filter diameter at expansion (mm)	Guidewire compatibility (inch)	Length (cm)
Nipro	Filtrap	3.5	0.014	180
		5	0.014	180
		6.5	0.014	180
		6.5	0.014	300
		8	0.014	180
		8	0.014	300
Tri-Med	Parachute	5	0.014	190
		5	0.014	270
		6.5	0.014	190
		6.5	0.014	270
		8	0.014	270
		8	0.014	50
		8	0.014	190

### Recommendations

Distal protection can be considered in selective cases when plaque burden is large and there is a high possibility of distal embolism or no reflow or cases with myocardial infarction in saphenous vein grafts.

f. Pharmacological intervention for no reflow

In 2017 ESC guidelines [20], using GP IIb/IIIa inhibitors as bail-out therapy is considered as class IIa indication in the event of angiographic evidence of a large thrombus, slow or no reflow, although this strategy has not been tested in a randomized trial.

### Evidence from Japan

Ishii et al. performed a randomized trial among 368 STEMI patients undergoing primary PCI [the nicorandil group (*n* = 185) or control group (*n* = 183)] [91]. They reported that intravenous 12 mg of nicorandil before primary PCI significantly improved ST-segment resolution and epicardial coronary flow, resulting in preventing cardiovascular events of long duration and deaths, compared to placebo group.

Miyazawa et al. studied the effect of nicorandil in STEMI, randomizing patients with STEMI to the nicorandil group (*n* = 35) or control group (*n* = 35) [92]. In the nicorandil group, 2 mg of nicorandil was injected directly into the infarct area prior to reperfusion by PCI. With nicorandil infusion, additional ST elevations without chest pain were observed for a few minutes in 94% of cases. However, no ventricular fibrillation or ventricular tachycardia occurred. TIMI grade 3 rates were significantly higher in the nicorandil group (40% vs. 17%, *p* < 0.01). Rates of adverse events were similar, however, left ventricular regional wall motion score significantly improved in the nicorandil group (*p* < 0.05). The effect of nicorandil was pronounced in patients without ischemic preconditioning.

Kobatake et al. compared the effects of nitroprusside (*n* = 25) with nicorandil (*n* = 24) on the slow/no-reflow phenomenon during primary PCI [93]. The degree of improvement in TIMI flow grade (post–pre/pre) and TIMI frame count (pre-post/pre) showed that nitroprusside was more effective than nicorandil (nitroprusside vs. nicorandil: 0.88 ± 0.79, 0.37 ± 0.37, *p* = 0.008; 0.59 ± 0.23, 0.36 ± 0.27, *p* = 0.003, respectively). At 1 year, rate of MACE was not significantly different (5/25 vs. 9/24, *p* = 0.175).

Further studies are needed to determine optimal methods of administration and doses of nicorandil because nicorandil has dose-dependent effects on coronary artery diameters and coronary blood flow.

More recently, a network meta-analysis was published comparing the effect of 7 intracoronary agents (adenosine, anisodamine, diltiazem, nicorandil, nitroprusside, urapidil, and verapamil) on the no-reflow phenomenon in patients with STEMI undergoing primary PCI, including 41 randomized control trials with 4,069 patients [94]. Anisodamine (α1 adrenergic receptor antagonist used in the treatment of acute circulatory shock in China) was associated with improved post-procedural TIMI flow grade, more occurrences of ST-segment resolution, and improvement of LVEF. The cardioprotective effect of anisodamine conferred a MACE-free survival benefit. Additionally, nitroprusside was regarded as efficient in improving coronary flow and clinical outcomes. Compared with standard care, adenosine, nicorandil, and verapamil improved coronary flow but had no corresponding benefits on cardiac function and clinical outcomes.

Considering GP IIb/IIIa inhibitors and anisodamine are not available in Japan, use of nicorandil or nitroprusside prior to reperfusion by primary PCI may be considered reasonable.

### Recommendations

Intravenous nicorandil may be considered for STEMI patients before primary PCI within 12 h after symptom onset to prevent coronary microvascular impairment.

Intracoronary injection of nicorandil can be considered to bail out in case of slow flow or no reflow.

g. Direct stenting

Evidence in favor of direct stenting (stenting without predilation) in patients with STEMI comes from several studies [95]. Loubeyre et al. [96] randomized 206 patients with STEMI to direct stenting or stent implantation after balloon predilation. The composite angiographic (corrected TIMI frame count, slow flow/no reflow or distal embolization) endpoint (11.7% vs. 26.9%; *p* = 0.01) and ST-segment resolution (79.8% vs. 61.9%; *p* = 0.01) were better among patients randomized to direct stenting than among those randomized to stent implantation after predilation [96]. In the Harmonizing Outcomes with Revascularization and Stents in Acute Myocardial Infarction (HORIZONS-AMI), direct stenting (*n* = 698) compared with conventional stenting after predilation (*n* = 1830) was associated with better ST-segment resolution at 60 min after the procedure (median: 74.8% vs. 68.9%; *p* = 0.01) and lower 1-year rates of all-cause mortality (1.6% vs. 3.8%; *p* = 0.01) and stroke (0.3% vs. 1.1%; *p* = 0.049) [97]. The EUROTRANSFER Registry that included 1,419 patients showed that direct stenting (*n* = 276) was superior to stenting after predilation in terms of post-procedural TIMI flow grade of 3 (94.9% vs. 91.5%; *p* = 0.02), no reflow (1.4% vs. 3.4%; *p* = 0.035), ST-segment resolution of > 50% (86.2% vs. 76.3%; *p* = 0.016) and 1-year mortality (2.9% vs. 6.5%; *p* = 0.047 after adjustment for propensity score) [98]. Direct stenting may be advantageous over stenting after predilation in several aspects including the use of fewer and shorter stents, shorter fluoroscopy time and less use of contrast media and reduced microvascular dysfunction/obstruction and no reflow by reduced distal embolization. Potential disadvantages of direct stenting may include: failure to reach and/or to cross the lesion, stent loss, erroneous estimation of stent length, difficulty with stent positioning (especially in cases of persistent TIMI flow 0–1), underexpansion of the stent in an undilatable (i.e., calcified) lesion and stent undersizing due to underestimation of vessel diameter because of reduced flow [99]. Notwithstanding these disadvantages, direct stenting is now considered as acceptable alternative strategy as compared to conventional stenting during primary PCI.

### Recommendations

Direct stenting is recommended in primary PCI if possible.

h. Balloon angioplasty

The clinical efficacy of balloon angioplasty for STEMI is limited due to the relatively high percentage of restenosis caused by elastic recoil and late negative remodeling [100]. Several studies showed the need for repeat revascularization was significantly reduced by the use of coronary stents [101–103]. There is also Japanese evidence supporting this fact in patients with AMI [104, 105]. Nonetheless, stent implantation did not result in lower rates of recurrent MI or death, when compared with balloon angioplasty alone. Subsequently, numerous randomized trials demonstrated a further reduction in target lesion revascularization (TLR) could be achieved when using drug-eluting stents (DES) as opposed to bare-metal stents (BMS). Equivalent to studies comparing balloon angioplasty with stenting, though, none of these studies demonstrated a reduction in recurrent MI or death [106–108]. An important limitation of stent usage is a persistent risk of stent thrombosis (ST) and/or in-stent restenosis even years after implantation, particularly in patient subsets as STEMI [109–114].

Considering stent implantation may even induce no reflow and thereby expand infarct size [115–117], it may be reasonable to refrain from stenting if coronary flow is restored and no significant stenosis persists after thrombus aspiration and balloon dilatation. Indeed, recent studies have demonstrated it is safe to defer stent implantation in the acute phase of STEMI [118, 119]. Considering the absence of superiority with regard to hard clinical end points and the potential short- and long-term disadvantages of stent implantation, angioplasty with a drug-coated balloon (DCB) without stenting may well serve as a therapeutic strategy of choice in STEMI.

The PAPPA pilot study was the first prospective clinical trial studying the efficacy and safety of a DCB only strategy in PPCI for STEMI [120]. Additional stenting was allowed only in case of type C to F coronary dissection or residual stenosis > 50%. All patients were treated with intravenous bivalirudin. Of 100 consecutive STEMI patients, 59 patients were treated with a DCB only strategy, whereas bail-out stenting was required in 41 patients. At 1-year, five major adverse cardiac events were reported (5%). Cardiac death was seen in two patients, while three patients underwent TLR. Although in this pilot study the rate of bail-out stenting was relatively high, the use of a DCB angioplasty-only strategy in the setting of primary PCI seems to be a safe and feasible treatment modality.

The REVELATION trial randomized 120 patients presenting with STEMI either to treatment with a DCB (*N* = 60) or DES (*N* = 60) [121]. No death or recurrent MI was reported, and TLRs were performed in 2 patients of the DCB group and 1 patient in the DES group. The functional assessment of the infarct-related lesion by FFR at 9 months after initial treatment was performed in 34 and 39 patients in the DCB and DES groups, respectively, and their FFR values were similar (0.92 ± 0.05 versus 0.91 ± 0.06; *p* = 0.27). These results might suggest that angioplasty with a DCB without stenting could be a therapeutic strategy of choice in STEMI, although larger randomized trial is necessary to confirm the safety and efficacy of a DCB without stenting.

In the INNOVATION study, 114 patients receiving primary PCI for STEMI were randomized into deferred stenting (DS) or immediate stenting (IS) [122]. In the DS group, the primary procedures included thrombus aspiration and balloon angioplasty and the second-stage stenting procedure was scheduled to be performed at 3 to 7 days after primary reperfusion procedure. DS did not significantly reduce infarct size (15.0% versus 19.4%; *p* = 0.112) and the incidence of microvascular obstruction (MVO; 42.6% versus 57.4%; *p* = 0.196), compared with IS. However, in anterior wall myocardial infarction, infarct size (16.1% versus 22.7%; *p* = 0.017) and the incidence of MVO (43.8% versus 70.3%; *p* = 0.047) were significantly reduced in the DS group.

### Recommendations

Currently, primary PCI using a balloon-only strategy is not recommended over direct stenting.

i. IVUS/ OCT/ OFDI

### Pre-procedural IVUS/ OCT/ OFDI

In ESC guidelines for myocardial revascularization [123], intravascular imaging is recommended only in cases of restenosis and stent thrombosis to detect stent-related mechanical problems and to assess and guide PCI in the left main stem (IIa). According to the expert consensus document of the European Association of Percutaneous Cardiovascular Interventions (EAPCI) [124], when a culprit lesion attributable to a NSTE-ACS presentation is not evident angiographically, an intravascular imaging-based assessment to guide appropriate management should be considered. Thrombus detection, for which OCT/ OFDI is the current gold standard, facilitates identification of an ACS culprit lesion.

### Identification of culprit lesion

Optical coherence tomography (OCT), optical frequency domain imaging (OFDI) and intravascular ultrasound (IVUS) detect plaque ruptures in about half of ST-elevation myocardial infarction. However, the superior resolution and obligatory flushing with OCT sharply outlines the rupture cavity and residual fibrous cap fragment to optimize ruptured plaque identification. de Feyter and Ozaki previously demonstrated plaque rupture and thrombus were more frequently found in ACS than those with stable angina by angioscopy, while IVUS failed to discriminate unstable from stable plaque [125]. More recently, Kubo et al. reported, when compared with the gold standard of angioscopy, OCT can identify thrombus better than IVUS and differentiate between red and white thrombus although red thrombus can shadow and obscure underlying plaque morphology [75].

While pathological studies reported that plaque erosion plays a role in ACS, there was no clear OCT definition of plaque erosion previously. While Ozaki and his colleagues proposed that OCT-derived intact fibrous cap (IFC-ACS) can be plaque erosion for the first time, contrary to ruptured fibrous cap (RFC-ACS), distinct culprit lesion characteristics associated with IFC-ACS mechanisms are not identified by CT angiography or IVUS [74]. OCT has been used to monitor changes in thrombus burden when lesions are treated with thrombus aspiration or with pharmacotherapy [126, 127]. Prati et al. demonstrated in the CLIMA study that the simultaneous presence of four high-risk OCT plaque features [MLA < 3.5 mm^2^, FCT < 75 μm, lipid arc circumferential extension > 180°, OCT-defined macrophages] was found to be associated with a higher risk of major coronary events in 1,003 patients undergoing OCT evaluation of the untreated proximal LAD [128].

In addition, combined IVUS and Near-Infrared Spectroscopy (NIRS) imaging, in particular where an increased plaque burden and lipid component present, is able to differentiate culprit lesions from non-culprit lesions with a high accuracy in STEMI [129, 130] and NSTEMI [131].

### Distal embolization or periprocedural myocardial infarction during stent implantation

TCFA not only cause plaque rupture and thrombosis but also contribute to myonecrosis during stenting. Findings associated with peri-myocardial infarction are greyscale IVUS-attenuated plaques, especially when the amount of attenuated plaque is large and begins closer to the lumen than to the adventitia; when large virtual histology-IVUS necrotic core or a virtual histology-thin-cap fibroatheroma or similar findings with integrated backscatter-IVUS (lipid) or iMap (necrotic core) are present; when an OCT-thin-cap fibroatheroma is present; when large lipid-rich plaques are detected by OCT or NIRS; or when plaque rupture is detected by IVUS or OCT [132, 133]. Furthermore, Ozaki and his colleagues reported that IB-IVUS-identified TCFA as well as OCT-verified TCFA were significant independent predictors of periprocedural myocardial infarction (PMI) after PCI [133]. However, the positive predictive value is poor and one trial [88] did not show superiority of distal protection when treating lipid-rich plaques. Conversely, the absence of these findings indicates a low probability of a peri-myocardial infarction with a high negative predictive value.

### Post-procedural lesion assessment, especially OCT/OFDI

Prati and his colleagues reported that a total of 1002 lesions (832 patients) were assessed. Appropriate OCT assessment was obtained in 98.2% of cases and revealed suboptimal stent implantation in 31.0% of lesions, with increased incidence in patients experiencing major adverse cardiac events (MACE) during follow-up (59.2% vs. 26.9%; *p* < 0.001). They concluded that suboptimal stent deployment defined according to specific quantitative OCT criteria was associated with an increased risk of MACE during follow-up in CLI-OPCI II study [134]. Prati and his coworkers also indicated that in ACS patients undergoing PCI, a composite of OCT-defined suboptimal stent implantation characteristics at the culprit lesion and residual intrastent plaque/thrombus protrusion was associated with adverse outcome in CLI-OPCI ACS substudy [135]. Post-procedural assessment especially OCT appears to confer a favorable long-term clinical outcome in patients with ACS.

### Recommendations

IVUS/ OCT/ OFDI should be considered to detect stent-related mechanical problems.

IVUS can be used to assess severity and optimize treatment of unprotected left main lesions.

Post-procedural OCT/ OFDI assessment including presence of dissection, degree of incomplete stent apposition, and presence of thrombus protrusion and may contribute to reducing MACE in long-term follow-up.

j. Stent

### Drug-eluting stents

Some meta-analyses suggested the safety and efficacy of second-generation DES in STEMI patients. In a network meta-analysis of patients with STEMI undergoing primary PCI (12,453 patients from 22 trials) [136], CoCr-EES were associated with significantly lower rates of cardiac death or MI and ST than BMS. CoCr-EES was also associated with significantly lower rates of 1-year stent thrombosis (ST) than paclitaxel-eluting stents (PES). Sirolimus-eluting stents (SES) were also associated with significantly lower rates of 1-year cardiac death/myocardial infarction than BMS. CoCr-EES, PES, and SES, but not zotarolimus-eluting stents, had significantly lower rates of 1-year target vessel revascularization (TVR) than BMS, with SES also showing lower rates of TVR than PES. Another network meta-analysis with longer follow-up data analyzed twelve trials with 9,673 patients [137]. Second-generation DES were associated with significantly lower incidence of definite or probable ST (OR 0.59, 95% CI 0.39–0.89), MI (OR 0.59, 95% CI 0.39–0.89), and TVR at 3 years (OR 0.50: 95% CI 0.31–0.81) compared with BMS. In addition, there was a significantly lower incidence of MACE with second-generation DES versus BMS (OR 0.54, 95% CI 0.34–0.74) at 3 years. In a patient-level network meta-analysis in patients with STEMI undergoing primary PCI with a median follow-up of 3 years (10,979 patients from 15 trials) [138], DES were superior to BMS with respect to cardiac death, reinfarction, or target lesion revascularization (TLR), and definite or probable stent thrombosis. Although second-generation DES did not significantly reduce cardiac death, reinfarction, or TLR, compared to first-generation DES (HR 0.98, 95% CI 0.79–1.21), second-generation DES were better than first-generation DES in the reduction of definite or probable stent thrombosis (HR 0.56, 95% CI 0.36–0.88).

In terms of long follow-up, recently, the EXAMINATION-EXTEND (10-Years Follow-Up of the EXAMINATION Trial) study demonstrated the superiority of CoCr-EES (*N* = 751) in combined patient- and device-oriented composite endpoints, compared with BMS (*N* = 747), in patients with STEMI (patient-oriented composite endpoint: 32.4% vs. 38.0%, HR 0.81, 95% CI 0.68–0.96, *p* = 0.013; device-oriented composite endpoint: 13.6% vs. 18.4%, HR 0.72, 95% CI 0.55–0.93, *p* = 0.012, respectively) [139]. These results were driven mainly by TLR (5.7% vs. 8.8%; *p* = 0.018). The rate of definite stent thrombosis was similar in both the groups (2.2% vs. 2.5%; *p* = 0.590). No differences were found between the groups in terms of target lesion revascularization (1.4% vs. 1.3%; *p* = 0.963) and definite or probable stent thrombosis (0.6% vs. 0.4%; *p* = 0.703) between 5 and 10 years.

The efficacy of a new-generation ultrathin strut DES, Orsiro, was demonstrated in the BIOSTEMI trial [140]. In this trial, 1,300 STEMI patients were enrolled, and the primary endpoint of target lesion failure (TLF: cardiac death, target vessel MI, and clinically indicated TLR) at 1 year was 4% with Orsiro and 6% with Xience (RR 0.59, 95% CI 0.37–0.94).

Overall, use of new-generation DES is encouraged, although the clinical benefit of ultrathin strut DES should be further investigated.

### Drug-coated stents

The LEADERS-FREE (Prospective Randomized Comparison of the BioFreedom Biolimus A9 Drug-Coated Stent versus the Gazelle Bare-Metal Stent in Patients at High Bleeding Risk) study compared the polymer-free biolimus-eluting Biofreedom stent with a bare-metal stent (BMS) in a cohort (*N* = 2466) at high risk of bleeding. In a subgroup analysis of 659 ACS patients, treatment with the BioFreedom stent remained more effective (clinically driven target lesion revascularization 3.9% vs. 9.0%, *p* = 0.009) and safer (cumulative incidence of cardiac death, MI, or definite or probable stent thrombosis 9.3% vs. 18.5%, P = 0.001), driven by significantly lower rates of cardiac mortality (3.4% vs. 6.9%, *p* = 0.049) and MI (6.9% VS 13.8%, *p* = 0.005) [141].

These results confirm the clinical utility of the drug-coated stents for patients at high bleeding risk and a direct comparison with current generation DES would be of great interest.

### Evidence from Japan

There are scarce randomized studies comparing between stents in Japan. Sawada et al. randomized patients with STEMI to receive EES (*n* = 23) or SES (*n* = 12) and compared arterial healing by OCT [142]. Both the EES and SES showed an excellent suppression of neointimal proliferation in the culprit lesion. The frequency of uncovered and malapposed struts of EES was significantly lower than that of SES (2.7% vs. 15.7%, *p* < 0.0001, 0.7% vs. 2.3%, *p* < 0.0001, respectively). EES may promote better arterial healing response than SES in patients with STEMI. The NAUSICA trial randomized patients with STEMI to Nobori biolimus A9 eluting stent (BES) or BMS and aimed to compare MACE at 1 year. However, the main results have not yet been published.

### Recommendations

- Stenting with recent generation DES is recommended over BMS for primary PCI.

k. Post-procedural IVUS/ OCT/ OFDI

Post-procedural IVUS/ OCT/ OFDI is used to evaluate stent underexpansion, malapposition, tissue protrusion, dissection, geographic miss, and thrombus. In the expert consensus document of the EAPCI [143], a relative stent expansion of > 80% (minimal stent area [MSA] divided by average reference lumen area), and an MSA of > 5.5 mm^2^ by IVUS and > 4.5 mm^2^ by OCT in non-left main lesions are recommended.

In the ULTIMATE trial [144], 1,448 patients were randomized to IVUS versus angiographic guidance. IVUS guidance was associated with a lower target vessel failure rate of 2.9% versus 5.4% (*p* = 0.019) at 1 year. In the IVUS-XPL trial [145, 146], 1,400 patients with long lesions were randomized to IVUS versus angiographic guidance. IVUS guidance was associated with a lower MACE rate of 5.6% versus 10.7% (*p* = 0.001) at 5 years. In CLI-OPCI observational study (n = 670), OCT guidance was associated with a significantly lower risk of cardiac death or MI as compared to angiographic only guidance [adjusted OR = 0.49 (0.25–0.96), *p* = 0.037]. Intravascular imaging-guided PCI has a potential to reduce cardiac death, major adverse cardiac events, stent thrombosis, and target lesion revascularization as compared with angiography-guided PCI [147]. OCT-guided PCI is noninferior to IVUS-guided PCI in terms of stent expansion in the ILUMIEN III trial [148] and clinical outcome in the OPINION trial [149] from Japan.

In general, a small edge dissection found on OCT which is undetected on angiography most likely does not have a clinical impact [150–153]. However, the following factors need to be considered: longitudinal and circumferential extension of dissection, and the depth of dissection (intima, media or even adventitia). In the ILUMIEN III [148], edge dissections were categorized as major if they constituted ≥ 60 degrees of the circumference of the vessel at the site of dissection and/or were ≥ 3 mm in length. In that trial, when the intra-dissection lumen area is < 90% of the respective reference area, additional stent implantation was considered. In CLI-OPCI-II trial [134], dissection was defined on OCT as a linear rim of tissue with a width of ≥ 0.2 mm and a clear separation from the vessel wall or underlying plaque. In this retrospective multicenter registry, acute dissection in the distal stent edge was an independent predictor for major adverse cardiac events.

If the malapposition distance from the endoluminal lining of strut to the vessel wall is < 250 µm, such struts likely come into contact with vessel wall at follow-up. Therefore, such small malappositions may be less clinically relevant [154, 155]. The clinical relevance of acute malapposition on stent failure is not yet fully established [134, 156–158]. Ozaki et al. reported that acute strut malapposition could persist (persistent malapposition; 4.67%), or resolve at follow-up (resolved/healed malapposition; 2.48%), whereas strut malapposition could also develop during follow-up (late acquired malapposition; 0.37%) [159]. The temporal evolution and disappearance of malapposition makes the investigation of the clinical relevance of strut malapposition more complicated.

### Recommendations

IVUS/ OCT/ OFDI can be used to optimize stent implantation.

A relative stent expansion of > 80% (MSA divided by average reference lumen area), and an MSA of > 5.5 mm^2^ by IVUS and > 4.5 mm^2^ by OCT in non-left main lesions should be achieved.

Acute incomplete stent apposition with a distance of ≤ 250 micron is likely to be resolved at follow-up. Additional post-dilatation is considered when malapposition distance is > 250 micron.

Most edge dissection detected on OCT is clinically silent, whereas additional stenting may be performed if the width of distal edge dissection is ≥ 200 micron [134].

l. Mechanical hemodynamic support

Intra-aortic balloon pumping (IABP) counterpulsation is the most widely used mechanical support for the treatment of cardiogenic shock, based on the beneficial effect of aortic diastolic inflation and rapid systolic deflation, improving myocardial and peripheral perfusion and reducing afterload and myocardial oxygen consumption.

The previous ESC guidelines stated that IABP may be considered in cardiogenic shock after STEMI (IIb) [22]. However, IABP counterpulsation does not improve outcomes in patients with STEMI and cardiogenic shock without mechanical complications [160, 161], nor does it significantly limit infarct size in those with potentially large anterior MIs [162]. The latest ESC guidelines no longer recommend routine IABP counterpulsation in cardiogenic shock except selected patients (i.e., severe mitral insufficiency or ventricular septal defect).

In other countries, mechanical LV assist devices (LVADs), including percutaneous short-term mechanical circulatory support devices (i.e., intra-cardiac axial flow pumps and arterial-venous extracorporeal membrane oxygenation) have been used in patients not responding to standard therapy, including inotropes, fluids, and IABP, but evidence regarding their benefits is limited [163]. A small exploratory trial studying the Impella CP percutaneous circulatory support device did not find any benefit compared with IABP in AMI complicated by cardiogenic shock [164]. Therefore, short-term mechanical circulatory support may be considered as a rescue therapy to stabilize patients and preserve organ perfusion (oxygenation) as a bridge to recovery of myocardial function, cardiac transplantation, or even LV assist device destination therapy on an individual basis [165, 166].

A structured approach to determine the best adjunctive mechanical circulatory support device requires understanding the mechanisms, technical requirements, and hemodynamic responses of each device [167] (Table 7). Device escalation is often required if the initial support device (usually IABP) does not improve hemodynamics and end-organ perfusion. Venoarterial extracorporeal membrane oxygenation (VA-ECMO) is often used in a combination with IABP to reduce the afterload increased by the retrograde flow. In a retrospective cohort study using propensity score matching in the Japanese Diagnosis Procedure Combination national inpatient database [168], all-cause 28-day mortality and in-hospital mortality were significantly lower in the IABP combined with VA-ECMO group than the VA-ECMO-alone group (48.4% vs 58.2%; *p* = 0.001 and 55.9% vs 64.5%; *p* = 0.004, respectively). The proportion of patients weaned from VA-ECMO was significantly higher in the IABP combined with VA-ECMO group than in the VA-ECMO-alone group (82.6% vs 73.4%; *p* < 0.001).

**Table 7:** Comparison of mechanical circulatory support system

	IABP	IMPELLA	VA-ECMO
Cardiac flow	0.3–0.5 L/min	1–5 L/min (Impella 2.5, Impella CP, Impella 5)	3–7 L/min
Mechanism	Aorta	LV → Ao	RA → Ao
Maximum implant days	Weeks	7 days	Weeks
Sheath size	7–8 Fr	13–14 Fr Impella 5.0—21 Fr	14–16 Fr arterial 18–21 Fr venous
Femoral artery size	> 4 mm	Impella 2.5 and CP: 5–5.5 mm Impella 5: 8 mm	8 mm
Cardiac synchrony or stable rhythm	Yes	No	No
Afterload	↓	↓	↑↑↑
Mean arterial pressure	↑	↑↑	↑↑
LVEDP	↓	↓↓	⟷
PCWP	↓	↓↓	⟷
LV preload	–	↓↓	↓
Coronary perfusion	↑	↑	–
Myocardial oxygen demand	↓	↓↓	⟷

Modified from [167]

*Ao* aorta, *IABP* intra-aortic balloon pump, *LA* left atrium, *LV* left ventricle, *LVEDP* left ventricular end diastolic pressure, *RA* right atrium, *PCWP* pulmonary capillary wedge pressure, *VA-ECMO*  venoarterial extracorporeal membrane oxygenation

There have been several clinical reports suggesting the combined use of Impella with IABP [169, 170]. However, this combination may decrease Impella forward flow during diastole due to diastolic pressure augmentation from the IABP [171].

The ongoing STEMI DTU (ST-elevation myocardial infarction door-to-unloading) trial (NCT03947619) will compare primary left ventricular unloading by Impella and a 30-min delay to reperfusion vs current standard of care in reducing infarct size and heart failure-related clinical events in patients presenting with anterior STEMI. The STEMI DTU trial will demonstrate whether Impella unloading of the left ventricle prior to reperfusion therapy reduces infarct size and thereby improves the prognosis of high-risk STEMI patients.

The latest guidelines for ACS from Japanese Circulation Society recommended IABP use as class I with level of evidence C [172], considering that percutaneous LVADs are not broadly available in Japan. However, the Impella 2.5 and Impella 5.0 heart pumps received Pharmaceuticals and Medical Devices Agency (PMDA) approval from the Japanese Ministry of Health, Labor & Welfare (MHLW) in September 2016 and received reimbursement, effective as of September 2017. Proper selection of patients and institutional criteria are being reviewed in J-PVAD (http://j-pvad.jp), and Impella has now been introduced in approximately 200 sites in Japan.

### Recommendations

Routine intra-aortic balloon pumping is not indicated.

Intra-aortic balloon pumping should be considered in patients with hemodynamic instability/cardiogenic shock due to mechanical complications.

In patients presenting refractory shock, short-term mechanical support (Impella or ECMO) may be considered in selected institutes.

m. DAPT in maintenance phase

### Risk stratification for bleeding

The PRECISE-DAPT score (age, creatinine clearance, hemoglobin, white-blood-cell count, and previous spontaneous bleeding) was derived from 14,963 patients treated with different durations of DAPT (mainly aspirin and clopidogrel) after coronary stenting and showed a c-index for out-of hospital TIMI major or minor bleeding of 0.73 (95% CI 0.61–0.85) [173]. A longer DAPT duration significantly increased bleeding in patients at high risk (score ~ 25), but did not in those with lower bleeding risk profiles, and exerted a significant ischemic benefit only in this latter group. As stated in the new ESC/EACTS Consensus document on DAPT, the use of risk scores such as PRECISE-DAPT designed to evaluate the benefits and risks of different DAPT durations ‘may be considered’ to support decision making [174].

Yoshikawa et al. reported that, in a pooled cohort of three studies conducted in Japan (12,223 patients from the CREDO Kyoto registry cohort-2, RESET and NEXT), the DAPT score successfully stratified ischemic and bleeding risks, although the ischemic event rate was remarkably low even with high-DAPT score [175].

### DAPT duration

Recent trials demonstrated the safety and efficacy of short DAPT followed by P2Y12 inhibitor monotherapy in ACS patients.

In the GLOBAL LEADERS trial, 1-month DAPT followed by ticagrelor monotherapy (experimental group) and 12-month DAPT (reference group) were compared [176]. In 7,487 patients with ACS, the primary outcome of death or new Q wave MI occurred in 55 patients (1.5%) in the experimental group and in 75 patients (2.0%) in the reference group between 31 and 365 days after randomization (HR 0.73, 95% CI 0.51–1.03; *p* = 0.07) [177]. BARC 3 or 5 bleeding happened in 28 patients (0.8%) in the experimental group and in 54 patients (1.5%) in the reference arm (HR 0.52, 95% CI 0.33–0.81; *p* = 0.004). These findings suggested that between 1 and 12 months after PCI in ACS, aspirin was associated with increased bleeding risk and appeared not to add to the benefit of ticagrelor on ischemic events. In the SMART-CHOICE trial [178], 1498 patients were randomized to either DAPT for 3 months followed by P2Y12 inhibitor (clopidogrel, prasugrel, or ticagrelor) monotherapy or DAPT for 12 months, in which 314 STEMI and 469 NSTEMI patients were included. The rate of BARC 2–5 bleeding was significantly lower in the P2Y12 inhibitor monotherapy group than in the DAPT group (2.0% vs 3.4%, HR 0.58, 95% CI 0.36–0.92, *p* = 0.02), and MACE rates were similar (2.9% vs 2.5%). The TWILIGHT trial examined the effect of ticagrelor alone after 3-month DAPT vs. ticagrelor plus aspirin among patients at high risk for bleeding or ischemic events after PCI [179]. Among patients with NSTE-ACS (*n* = 4614), ticagrelor monotherapy reduced BARC 2, 3, or 5 bleeding by 53% (3.6% vs. 7.6%, HR 0.47, 95% CI 0.36–0.61, *p* < 0.001). Rates of all-cause death, MI, or stroke were similar (4.3% vs. 4.4%, HR 0.97, 95% CI 0.74–1.28, *p* = 0.84) [180]. The TICO trial also compared ticagrelor monotherapy after 3-month DAPT vs. 12-month DAPT [181]. In 1,103 STEMI patients, ticagrelor monotherapy significantly reduced TIMI major bleeding (HR 0.32, 95% CI 0.12–0.87) without significant increase of MACE (HR 1.10, 95% CI 0.53–2.27). In 1,027 NSTEMI patients, ticagrelor monotherapy tended to reduce TIMI major bleeding (HR 0.69, 95% CI 0.34–0.143) and MACE (HR 0.58, 95% CI 0.30–1.13) [182]. These results corroborate the potential benefit of ticagrelor monotherapy after short DAPT in ACS patients.

Regarding the comparison between potent P2Y12 inhibitors, the ISAR-REACT 5 trial compared prasugrel plus aspirin vs. ticagrelor plus aspirin in ACS patients, and demonstrated that treatment with prasugrel, compared to ticagrelor, significantly reduced the composite rate of death, MI, or stroke (6.9% vs. 9.3%, *p* = 0.006) without any increase in bleeding complications (4.8% vs. 5.4%, *p* = 0.46) [55].

Recently, MASTER DAPT trial compared with 1-month DAPT and at least 6-month for patients without anticoagulation (at least 3-month for patients with anticoagulation) in high bleeding risk population, in which ACS patients were included. The rates of both net adverse clinical events (NACE) and major adverse cardiac or cerebrovascular events (MACCE) were similar (7.5% vs 7.7% and 6.1% vs 5.9%) and met the trial definition for non-inferiority. However, the rate of major and clinically relevant nonmajor bleeding was significantly lower in the abbreviated 1-month DAPT group, compared to the prolonged DAPT group (6.5% vs 9.4%, *p* < 0.001) [183, 184].

It is well known that aspirin induces gastrointestinal ulceration and erosion [185]. In the Management of Aspirin-induced Gastrointestinal Complications (MAGIC) study, patients receiving PPI had lower risk of gastrointestinal ulcer or erosion [186, 187] Therefore, PPI should be more constantly used in patients with aspirin to reduce gastrointestinal toxicity during long-term prevention of cardiovascular events.

### DAPT dosage

Both prasugrel and ticagrelor are available, but the dose of prasugrel is different in Japan. While 60 mg loading dose and 10 mg maintenance dose are applied in Europe and US, 20 mg loading dose and 3.75 mg maintenance dose are used in Japan. Although clopidogrel is dominantly used around the world, smaller dose of prasugrel including loading conveys less bleeding events associated without increase of ischemic events in Japan [52] (Table 1).

### Evidence from Japan

The STOP-DAPT 2 trial randomized 3,045 patients either to 1-month of DAPT followed by clopidogrel monotherapy or 12 months of DAPT with aspirin and clopidogrel [188]. One-month DAPT was superior to 12-month DAPT for the primary end point of all-cause death and new Q-wave MI, occurring in 2.36% with 1-month DAPT and 3.70% with 12-month DAPT (HR 0.64, 95% CI 0.42–0.98, *p* = 0.04). TIMI major or minor bleeding occurred in 0.41% with 1-month DAPT and 1.54% with 12-month DAPT (HR 0.26, 95% CI 0.11–0.64, *p* = 0.004). The results of the STOP-DAPT 2 ACS trial were presented in the ESC congress 2021, in which randomized 4,169 ACS patients with the same antiplatelet therapy regimen as the STOP-DAPT 2 trial were analyzed. The same primary endpoint as the STOP-DAPT 2 trial were applied to the ACS patients, and cumulative event rates were 3.2% in the 1-month DAPT group and 2.83% in the 12-month group, which did not meet the statistical significance for non-inferiority (HR 1.14, 95% CI 0.80–1.62, p non-inferiority 0.06), although cumulative rates of TIMI major and minor bleeding were significantly lower with 1-month DAPT (0.54% vs 1.17%, HR 0.46, 95% CI 0.23–0.94).

The STOP-DAPT-3 (NCT04609111) started for patients with high bleeding risk or ACS, in which 1-month prasugrel monotherapy followed by clopidogrel monotherapy (no aspirin) vs 1-month DAPT comprising of aspirin and prasugrel followed by aspirin monotherapy after PCI will be investigated. In terms of an aspirin-free strategy, the safety and efficacy of prasugrel monotherapy after PCI in selected patients with stable CAD and low ischemic and bleeding risk were investigated in ASET trial [189]. The primary ischemic and bleeding endpoints occurred in 1 patient (0.5%), and no stent thrombosis events occurred. To investigate the additional application of prasugrel monotherapy after PCI, the safety and efficacy of Japanese low-dose prasugrel monotherapy (3.5 mg) after PCI in patients with CCS (phase 1) and NSTE-ACS (phase 2) has been evaluated in 400 patients in the ASET-JAPAN trial (NCT05117866).

### Patients with atrial fibrillation

In patients with atrial fibrillation, after a short period of triple therapy up to 1 week from the acute event, 1-year combination therapy with direct oral anticoagulant (DOAC) and P2Y12 inhibitor, followed by DOAC/novel oral anticoagulant (NOAC) monotherapy could be recommended, but for high thrombotic risk patients, a period of triple therapy might be extended to 3–6 months [21, 190].

In patients with atrial fibrillation, the large 4 trials, WOEST, PIONEER AF, RE-DUAL PCI, and AUGUSTUS trials and their network meta-analysis demonstrate that the treatment with DOAC and P2Y12 inhibitor could reduce bleeding risk without an increased risk of ischemic events up to 1 year after PCI, compared to vitamin K-antagonist plus DAPT (i.e., triple therapy) [191–195]. In these 4 trials, approximately half of patients presented with ACS. Clopidogrel was used as a P2Y12 inhibitor in more than 90% patients. Recently, MASTER DAPT study comparing abbreviated and prolonged DAPT following Ultimaster stent™ implantation in high bleeding risk (HBR) patients indicated that abbreviated therapy resulted in a lower incidence of major or clinically relevant nonmajor bleeding[183]. Furthermore, a substudy of MASTER DAPT using clopidogrel in patients with oral anticoagulant (OAC) revealed that it is safe and beneficial to stop DAPT at 1 month in HBR patients with or without an indication for OAC, while an abbreviated antiplatelet therapy strategy significantly reduced clinically relevant bleeding risk in HBR patients without OAC but no such significant reduction was obtained in the OAC population [184].

The AFIRE trial demonstrated that DOAC monotherapy was noninferior to combination therapy with DOAC and single antiplatelet therapy for efficacy (stroke, systemic embolism, MI, unstable angina requiring revascularization, or all-cause death; HR 0.72, 95% CI 0.55–0.95) and superior for safety (major bleeding; HR 0.59, 95% CI 0.39–0.89) in patients with atrial fibrillation and stable coronary artery disuse including prior PCI more than 1 year earlier [196]. Although 4 major DOAC studies (i.e., WOEST, PIONEER AF, RE-DUAL PCI, and AUGUSTUS) clearly indicated the superiority of DOAC over warfarin, patients with impaired kidney function were exclude from such trial because DOAC are not recommended in patients with significant renal dysfunction. To address such real-world limitations, Ozaki and his colleagues performed REWRAPS study (NCT02024230) involving all comer patients regardless of kidney function. While all the patients had coronary stenting and AF in the REWRAPS study, 250 patients were assigned to Rivaroxaban and 245 patients were allocated to Warfarin associated with a minimum 3-year follow-up. Renal function appears to play a major role in prognosis of patients with impaired renal function. Furthermore, Hashimoto and Ozaki and his coworkers recently reported that 3-year mortality and MACE significantly deteriorated from 5.09% and 15.8% in no CKD through 16.3% and 38.2% in moderate CKD to 36.7% and 57.9% in severe CKD, respectively (*p* < 0.0001), based on 3,281 patients with AMI enrolled in the J-MINUET registry associated with primary PCI of 93.1% in STEMI [197]. They concluded that CKD remains a useful predictor of in-hospital and 3-year mortality as well as MACE after AMI in the modern PCI and optimal medical therapy era [197]. Recently, Collet JP and the task force for the management of ACS of the European Society of Cardiology (ESC) recommended in patients with atrial fibrillation and high bleeding risk, triple antithrombotic therapy with DOAC, aspirin, and clopidogrel should be given in a short period up to 1 week followed by double therapy using DOAC and clopidogrel for 6 months then DOAC monotherapy after the 6 months, while in those with atrial fibrillation and high ischemic risk, triple antithrombotic therapy including DOAC, aspirin, and clopidogrel should be provided up to 1 month followed by double therapy consisting of DOAC and clopidogrel for 12 months then DOAC monotherapy after the 12 months [21].

### Field of still lack of evidence in DAPT duration

While TWILIGHT, GLOBAL LEADERS, STOP-DAPT-2 and MASTER DAPT trials have proved the superiority of shorter DAPT strategy in various clinical setting, the scientific direction surely is moving towards to shorter and abbreviated DAPT duration to reduce bleeding events [176, 179, 183, 188]. Recently two new advance trials such as ASET Japan and STOP-DAPT-3 have launched to confirm “aspirin is only before stenting but not after stenting” strategy in chronic coronary syndrome and subsequently acute coronary syndrome. In ASET Japan patients was only enrolled with optimal stenting results confirmed by OCT/ OFDI/ IVUS. However, no trial has not yet done in comparison with shorter abbreviated and longer standard DAPT in bifurcation two stenting including LMT, fill mental jacket and a history of stent thrombosis. While imaging-guided stent optimization can be a solution to reduce the ischemic complications in such complex PCI in long-term follow-up, non-uniform DAPT duration should be considered to account for complexity such as bifurcation two stenting including LMT, fill mental jacket and stent thrombosis. However, even in such cases, shortening the DAPT duration should always be kept in mind to reduce bleeding events.

### Recommendations

Short DAPT (1 month) followed by a potent P2Y12 inhibitor (possibly prasugrel or ticagrelor) monotherapy should be considered after PCI in patients with high bleeding risk based on recent publications including the GLOBAL LEADERS, STOP-DAPT2 and MASTER DAPT trials.

One-month DAPT followed by clopidogrel monotherapy may be not recommended in patients with ACS.

Prolonged DAPT (at least 6 months) should only be considered for patients with high thrombotic risk such as patients with ACS or history of stent thrombosis as well as lesions with bifurcated two stenting or full mental jacket.

In patients with atrial fibrillation and high bleeding risk, triple antithrombotic therapy with DOAC, aspirin, and clopidogrel should be given in a short period up to 1 week followed by double therapy using DOAC and clopidogrel for 6 months, while in those with atrial fibrillation and high ischemic risk, triple antithrombotic therapy including DOAC, aspirin, and clopidogrel should be given up to 1 month followed by double therapy consisting of DOAC and clopidogrel for 12 months then DOAC monotherapy after the 12 months.

A proton pump inhibitor (PPI) in combination with DAPT is recommended in patients at high risk of gastrointestinal bleeding.

In patients with LV thrombus, anticoagulation should be administered for at least 6 months guided by repeated ultrasound or CT/MRI imaging.

## Treatment of non-infarcted-related artery

### General recommendation in revascularization of non-infarct-related artery in acute MI

In the guidelines released by the European Society of Cardiology in 2017 on the management of patients with ST-segment elevation MI Complete revascularization for ST-segment elevation MI patients with multivessel disease was upgraded from III to IIa with the level of evidence A.

In the Compare-Acute trial, 885 patients with ST-segment elevation MI and multivessel disease who underwent primary PCI were randomized in a 1:2 fashion to complete revascularization of non-infarct-related coronary arteries guided by FFR or no revascularization of non-infarct-related coronary arteries [198, 199]. There was a significant reduction in MACE at 3 years with FFR-guided complete revascularization (15.6% vs 30.2%, HR 0.46, 95% CI 0.33 to 0.64, *p* < 0.001). The benefit was mostly driven by a reduced risk of revascularization. In the COMPLETE trial, 4041 patients with ST-segment elevation MI and MVD who underwent primary PCI were randomized in a 1:1 fashion to complete revascularization of non-infarct-related coronary arteries guided by FFR or no revascularization of non-infarct-related coronary arteries [200]. At 5 years, FFR-guided complete revascularization significantly reduced cardiovascular death or MI (7.8% vs 10.5%, HR 0.74, 95% CI 0.60 to 0.91, *p* = 0.004). In a meta-analysis published in 2020, these trials are included, and complete revascularization had a benefit for cardiovascular death (odds ratio 0.69, 95% CI 0.48–0.99) [201].

In the setting of cardiogenic shock, the efficacy and safety of treating non-infarct-related coronary arteries in the context of primary PCI has been a matter of debate. In the CULPRIT-SHOCK (Culprit Lesion Only PCI versus Multivessel PCI in Cardiogenic Shock) trial (*N* = 706), the 30-day risk of a composite of death or severe renal failure leading to renal-replacement therapy was lower in patients who underwent initial PCI of the culprit lesion only compared with those who underwent immediate multivessel PCI [202]. Between 30 days and 1 year, there was no significant difference in all-cause death between the two groups [203].

In 2017 ESC guidelines, published 2 months before the CULPRIT-SHOCK trial, Grade IIa recommendation with level of evidence C was applied for complete revascularization in ST-segment elevation MI at patients with multivessel disease who present with cardiogenic shock. However, in 2018 ESC guidelines on myocardial revascularization [123], routine revascularization of non-IRA lesions is not recommended during primary PCI in patients with cardiogenic shock (Class III).

In 2020 ESC guidelines for NSTE-ACS, complete revascularization in NSTE-ACS patients without cardiogenic shock and with multivessel CAD is recommended as Class IIa [21]. Data from the British Cardiac Intervention Society PCI database showed significantly lower mortality rates with single-stage complete revascularization compared to culprit-lesion-only PCI (adjusted HR 0.90, 95% CI 0.85–0.97) at a median follow-up of 4.6 years among 21,857 NSTE-ACS patients with multivessel CAD undergoing PCI [204]. In the Alberta COAPT registry, complete revascularization significantly reduced all-cause death or new MI, compared to incomplete revascularization (inverse probability-weighted HR 0.78, 95% CI 0.73–0.84) in patients with ACS and MVD [205]. The significant reduction was observed regardless of ACS type (STEMII, NSTEMI, or unstable angina).

In terms of the timing of non-culprit lesion PCI, a substudy in the COMPLETE trial investigated the timing of non-culprit lesion PCI [206], and PCI during index hospitalization or after discharge conferred similar benefits on major cardiovascular events. The SMILE trial randomized 500 NSTE-ACS patients to immediate complete revascularization vs staged complete revascularization. Immediate complete revascularization significantly reduced MACE (13.6% vs 23.2%, HR 0.55, 95% CI 0.36–0.83) [207]. We still need dedicated randomized trials for the best timing of non-culprit lesion PCI.

### Recommendations

Complete revascularization should be considered in STEMI or NSTEMI patients with multivessel disease.

Non-IRA PCI during the index procedure is not recommended in patients with cardiogenic shock.

### Physiological assessment of non-infarct-related artery

The Compare-Acute and COMPLETE trials applied fractional flow reserve (FFR) guide assessment of non-infarcted-related artery for complete revascularization in STEMI patients, and in both trials, FFR-guide complete revascularization significantly reduced cardiovascular events, compared to culprit-lesion-only PCI.

Regarding the impact of physiological assessment for complete revascularization, 306 STEMI patients were randomized to angiography-guided or stress echo-guided PCI for non-infarct-related lesions in the CROSS-AMI [208]. The primary endpoint of cardiovascular mortality, nonfatal reinfarction, coronary revascularization, or readmission because of heart failure occurred in 14% in both groups (*p* = 0.85). However, this trial had a rather small sample size.

Direct comparison between angiography-guided and FFR-guided complete revascularization was performed in the FLOWER-MI study [209]. The primary outcome was a composite of death from any cause, nonfatal myocardial infarction, or unplanned hospitalization leading to urgent revascularization at 1 year, and a primary outcome event occurred in 32 of 586 patients (5.5%) in the FFR-guided group and in 24 of 577 patients (4.2%) in the angiography-guided group (HR 1.32, 95% CI 0.78–2.23; *p* = 0.31). Although an FFR-guided strategy failed to show a significant benefit over an angiography-guided strategy, considering the given the wide CIs for the estimate of effect we still need the evidence for physiological assessment of non-infarcted-related artery in acute setting.

### Summary (Fig. 1)

**Fig. 1:**
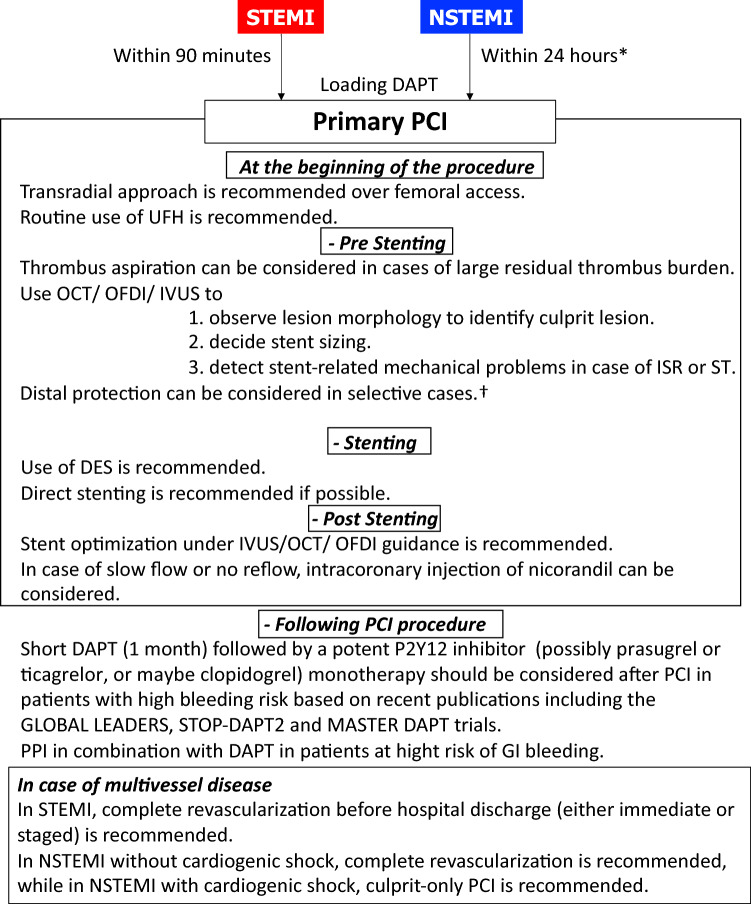
Summary of recommendations in primary PCI. *Urgent coronary angiography (< 2 h) is recommended in very high-risk patients. †Cases with large thrombus formation or plaque burden with a high possibility of distal embolism or slow/no flow; or cases with MI in SVG. *DAPT* dual antiplatelet therapy; *DES* drug-eluting stent; *GI* gastrointestinal; *ISR* in-stent restenosis; *IVUS* intravascular ultrasound; *NSTEMI* non-ST-segment elevation myocardial infarction; *OCT* optical coherence tomography; *PCI* percutaneous coronary intervention; *PPI* proton pump inhibitor; *ST* stent thrombosis; *STEMI* ST-segment elevation myocardial infarction; and *UFH* unfractionated heparin

The Task Force on Primary PCI of the CVIT society has updated this expert consensus document for the management of AMI in 2022 version based on the new evidence (Fig. 1). Our team would like to recommend the following strategies in STEMI: (1) primary PCI should be done within 90 min; (2) radial access and drug-eluting stent (DES) over bare-metal stent (BMS) are recommended; (3) complete revascularization before hospital discharge (either immediate or staged) is now preferred. For patients with NSTEMI, we also now recommend an early invasive strategy within 24 h, and complete revascularization in NSTEMI patients without cardiogenic shock.

Although in ESC guidelines [123] intravascular imaging is recommended only in case of restenosis and stent thrombosis to detect stent-related mechanical problems and to assess and guide PCI in left main stem, in the recent expert consensus document of the European Association of Percutaneous Cardiovascular Interventions (EAPCI) [124], an intravascular imaging-based assessment to guide appropriate management should be considered when a culprit lesion is not evident angiographically in NSTE-ACS. Furthermore, thrombus detection where OCT/ OFDI is the current gold standard facilitates identification of an ACS culprit lesion. IVUS/ OCT/ OFDI should be considered to detect stent-related mechanical problems. IVUS can be used to assess severity and optimize treatment of unprotected left main lesions. Post-procedural OCT/OFDI assessment including presence of dissection, degree of incomplete stent apposition, and presence of thrombus protrusion could contribute to reducing MACE in long-term follow-up.

Furthermore, although earlier studies have shown the benefit of thrombus aspiration in primary PCI published in New England Journal Medicine and European Heart Journal from the Dutch group [78, 79], routine use of mechanical thrombus aspiration is no longer recommended due to the safety concerns regarding the risk of stroke. However, in the subgroup with high thrombus burden, thrombus aspiration was associated with fewer cardiovascular deaths but with more strokes or transient ischemic attacks [77]. Futhermore, there are several studies in Japan showing the benefit of thrombus aspiration in primary PCI. Therefore, in the absence of GP IIb/IIIa inhibitors, careful thrombus aspiration may be considered in primary PCI, especially in patients with high thrombus burden.

Concerning the duration of antiplatelet therapy, short DAPT (1 month) followed by P2Y12 inhibitor has become the first choice in patients with high bleeding risk, however in the near future P2Y12 inhibitor monotherapy might be enough after PCI in patients without atrial fibrillation. Furthermore, although clopidogrel is dominantly used around the world, smaller dose of prasugrel including loading confers fewer bleeding complications associated without increased risk of ischemic events in Japan. In patients with atrial fibrillation and high bleeding risk, following a short period of triple antithrombotic therapy (up to 1 week from the acute event), dual antithrombotic therapy (e.g., DOAC and single oral antiplatelet agent preferably clopidogrel) is recommended with cessation of antiplatelet therapy after 6 months. In patients with atrial fibrillation and high ischemic risk, triple antithrombotic therapy including DOAC, aspirin, and clopidogrel should be given up to 1 month followed by double therapy consisting of DOAC and clopidogrel for 12 months then DOAC monotherapy after the 12 months.

While the Compare-Acute and COMPLETE trials applied FFR-guide assessment of non-infarcted-related artery for complete revascularization in STEMI patients, FFR-guide complete revascularization significantly reduced cardiovascular events compared to culprit-lesion-only PCI. However, the FLOWER-MI study performed a direct comparison between angiography-guided and FFR-guided complete revascularization and failed to show a significant benefit over an angiography-guided strategy. We still need the evidence for physiological assessment of non-infarcted-related artery in acute setting. Furthermore, in the near future, such physiological assessment will be preferred to be less invasive manner including QFR or FFR-CT especially in non-culprit vessels.
